# The influence of meteorological factors on the technical performance of football teams during matches

**DOI:** 10.5114/biolsport.2024.136091

**Published:** 2024-04-25

**Authors:** Yonghan Zhong, Shaoliang Zhang, Qing Yi, Miguel Ángel Gómez Ruano

**Affiliations:** 1 School of Athletic Performance, Shanghai University of Sport, Shanghai, China; 2 Division of Sports Science and Physical Education, Tsinghua University, Beijing, China; 3 College of Physical Education, Dalian University, Dalian, China; 4 Facultad de Ciencias de la Actividad Física y del Deporte (INEF), Universidad Politécnica de Madrid, Madrid, Spain

**Keywords:** Soccer, Team sport, Environmental effect, Technical performance, Match analysis

## Abstract

This study explored the impact of the meteorological factors air temperature, relative humidity, and wind speed on teams’ technical performance during the Union of European Football Associations Champions League (UCL). Technical match statistics were collected for five seasons (2016/2017–2020/2021). Thirty-one technical actions and events were categorized into three groups (goal scoring, offense, and defence). Meteorological data were collected from the official UEFA website (air temperature (°C): 11.59 ± 6.87, relative humidity (%): 71.40 ± 17.82, wind speed (km/h): 11.52 ± 7.02). LASSO regression analysis was employed to identify important indicators that affect match outcomes, while separate Poisson regression models were used in generalized linear modelling to determine the effects of meteorological factors on key technical performance indicators. The results revealed that offensive variables were instrumental in distinguishing match outcomes (winning, drawing, and losing), with meteorological factors significantly influencing these variables. Notably, “shot from counter attack” was the most significantly affected variable, being exclusively influenced by air temperature (OR = 0.55, 95% CI: 0.30 to 0.98). Two technical indicators, “dribbles won” and “shots from the 6-yard box,” were both significantly influenced by two meteorological factors. “Cards per foul,” an understudied variable, displayed a close association with all the three meteorological factors. These findings offer valuable insights for coaches and analysts in comprehending the influence of meteorological conditions on crucial technical variables during the performance evaluation of teams. Moreover, they provide valuable information to help coaches devise appropriate tactics for players before or during a match, considering the potential changes in meteorological conditions.

## INTRODUCTION

Factors affecting the performance of football teams are complex. Most previous studies have shown that a football team’s technical performance is affected by situational factors [1, 2]. The emergence of global warming in addition to situational factors has led researchers to increasingly focus on football players’ performance in different weather conditions to explore the key factors that enhance technical and physical performance in different playing meteorological conditions and to prevent sports injuries.

In previous studies, the wet bulb globe temperature (WBGT) has been widely used to explain meteorological factors. It assesses air temperature, relative humidity, wind speed, and solar radiation in its output [3]. However, d’Ambrosio Alfano et al. [4] recently critically reviewed the WBGT and clearly demonstrated that it is becoming obsolete. As with all indices that integrate elements of the thermal environment, measurement errors due to substandard instruments and poor calibration procedures are common. Due to its limitations, WBGT can provide only a general guide to the likelihood of adverse effects of heat [5]. Based on the above, choosing the four basic weather elements over the WBGT index is preferable [6]. In 2011, the proposal of a new heat stress index for outdoor thermal environments began to slowly replace the WBGT: the universal thermal climate index (UTCI) [7]. However, it has been argued that the UTCI is less suitable for assessing environments with high wind speeds [8]. To avoid potential errors in the process of calculating the index, this study evaluated three meteorological variables directly: air temperature, relative humidity, and wind speed.

The football match is played under complex meteorological conditions. Understanding the impacts of meteorological factors on the team’s technical performance may help coaches to prepare the upcoming matches better [9]. Many studies have shown that air temperature, relative humidity, and wind speed can have an impact on football technical performance. All the parameters related to technical performance change when relative humidity and the WBGT index increase by two standard deviations (SDs) [1]. A qualitative analysis by Nassis et al. (2015) demonstrated that successful passing, a technical performance indicator (PI), is significantly correlated with meteorological factors [10]. Grantham et al. (2010) stated that players are not at risk of heat stress when they play in environments below 22°C, are at low risk of heat stress when they play in 22–28°C environments, and at high risk when they play in environments above 28°C [11]. In addition, air temperature and relative humidity also affect the evaporation of sweat and hence body temperature, which subsequently affects the performance of players [12]. Zhou et al. explored the influence of the environment on the sports performance of Chinese Super League players. They found that to improve player performance, a comfort zone of air temperature (10.6 to 22°C) may exist and higher or lower air temperatures may affect player performance during football matches [13]. As relative humidity increases, an athlete’s exercise ability in a high-temperature environment gradually decreases, mainly due to the rapid increase of core and skin temperatures, which leads to premature entry into the fatigue stage [12].

Few studies have directly examined the influences of air temperature, relative humidity, and wind speed on team technical performance. Available literature has mainly focused on variance analysis by analysing the differences in game performance in different meteorological environments, but few researchers have performed predictive analysis. Predictive analysis can better predict risk and facilitate better decision making [14]. Therefore, such analysis results can provide more valuable references for team preparation.

Regarding indicator selection, few scholars have screened the PIs included in the analysis. A model with more regressors may reduce bias as well as the accuracy of predictions; therefore, choosing the most important variables is essential [15]. Consequently, the current study aimed to explore the impacts of meteorological variables (air temperature, relative humidity, and wind speed) on the technical performance of teams during UCL. Important features were selected from 31 technical performance variables. Including as many technical variables as possible will allow more accurate screening of variables in the future.

## MATERIALS AND METHODS

### Sample and variables

This paper explores the impact of meteorological factors on the technical performance of the whole team. Match statistics of teams in the Union of European Football Associations Champions League (UCL) from season 2016/2017 to 2020/2021 were collected from two sources; the data on technical performance and match outcomes were collected from “whoscored.com” (http://www.whoscored.com), whose original data from the company OPTA Sportsdata have been tested with a respectable level of inter-operator reliability (kappa values > 0.90) [16]. The data of air temperature, relative humidity, and wind speed for each match were retrieved from the official UEFA website (http://www.uefa.com). A total of 583 matches (1,166 observations) were selected as the sample based on available data on the official UEFA website after excluding matches without meteorological data. This included 1,166 sets of match outcomes. Thirty-one technical performance-related variables were included in the statistical modelling. Technical actions or events were classified into the following three groups (Table 1): (1) goal scoring, (2) offense, and (3) defence [17]. No human being or animal was involved in this study. Therefore, no written informed consent from the participants or ethical review was required.

**TABLE 1:** Selected technical performance-related match variables.

Groups	Variables
Variables related to goal scoring	Shot, Shot on target, Shot from open play, Shot from set piece, Shot from counterattack, Shot from 6 yards box, Shot from 18 yards box, Shot from outside of box
Variables related to offense	Dribble won, Dribble attempt, Dribble past, Dribble success, Corner, Corner accuracy, Accurate pass, Key pass, Pass success, Cross, Through ball, Long ball, Short ball, Possession, Aerial won, Touch
Variables related to defense	Tackle, Tackle attempt, Tackle success, Interception, Foul, Yellow card, Card per foul

### Data standardization

Before statistical analysis, data normalization for air temperature, relative humidity, and wind speed data was performed using the min-max method (linear normalization). This method’s key advantage is the ability to preserve the relationships between the initial data [18]. Equation (1) shows the formula for min-max normalization.

$$\text{Y}=({\text{X}}_{\text{original}}-{\text{X}}_{\min})/({\text{X}}_{\max}-{\text{X}}_{\min})$$ (1)

where Xoriginal is the original value of the independent variable, and X_min_ and X_max_ are the minimum and maximum values of the independent variables, respectively. The final adjusted value, Y, is obtained. The descriptive statistics of three meteorological variables are presented in Table 2.

**TABLE 2:** Descriptive statistics for Meteorological variables.

Variables	n	Mean	Standardized mean	Min.	Max.
Temperature (°C)	1166	11.6	0.53	-9	30
Humidity (%)	1166	71.4	0.7	5	100
Wind speed (km/h)	1166	11.5	0.24	1	29

### Procedure and statistical analysis

The present study employed the LASSO method to regularize and select features in the regression analysis; this method is reliable for variable selection by regularization, applicable to a large class of linear models. LASSO penalizes models by adding an L1 norm of the model parameters to the cost function. The L1 norm favours models with many coefficients set to 0, effectively performing feature selection by suppressing variables that have a small influence on the target variable, weak predictors correlated to stronger variables, and, in this particular case, variables for which it was not possible to decide on the direction of the effect on match outcome on the basis of the analysed data set [19]. In the LASSO, each regression coefficient is transformed using a constant parameter, λ. As λ increases, the regression coefficient related to the independent variable progressively diminishes and eventually reaches zero. Consequently, the selection of the optimal λ value becomes crucial, as it determines the significant variables with non-zero regression coefficients. The optimal λ value is determined via cross-validation, aiming to minimize the mean squared error (minMSE) or minMSE plus one standard error (minMSE + 1SE) [20].

The LASSO regression approach is employed to select influential feature variables that impact the match outcomes. The match outcomes were used as multi-categorical variable: 3 – win, 1 – draw, and 0 – loss. The technical performance-related variables were employed as the predictors, while the match outcomes were the dependent variables. Cross-validation was performed to obtain the best λ, the minMSE, and minMSE + 1SE values. The primary function employed for cross-validation was cv.glmnet. Notably, when λ equals the minMSE value, most variables exhibit a non-zero regression coefficient, encompassing a more comprehensive range of technical performance measures compared with the minMSE + 1SE scenario. Hence, the minMSE value was used to identify the feature variables with non-zero regression coefficient values, a task accomplished using the coef function. These aforementioned methods were implemented within the glmnet R package [21].

Subsequently, a generalized linear model (GLM) was employed to forecast the influence of air temperature, relative humidity, and wind speed on technical performance. The GLM was implemented using the glmnet function in R (RStudio 4.2.1 version). Distinct Poisson regression analysis was conducted within the model, designating each of the 15 chosen technical performance-related variables as the dependent variable. Meanwhile, three normalized meteorological variables were selected as the independent variables. Given that both teams in a football match are subjected to identical meteorological conditions, the impact of other internal factors pertaining to the teams (e.g., home and away, team ability, or opponent’s strength) is extensively mitigated when matches are analysed holistically. To estimate the specific influence of meteorological variables on technical performance-related variables, odds ratios (ORs) accompanied by 95% confidence intervals (CIs) were employed. The point estimate of the OR was deemed statistically significant at a P level below 0.05 if the 95% CI did not include 1 [22]. Notably, a 10% change corresponds to a 1SD increase in the air temperature, relative humidity, and wind speed variables, yielding a 10% likelihood of change in the technical performance-related variables. This change is considered as the minimum meaningful alteration in value [23, 24].

## RESULTS

### Indicator selection

**FIG. 1:**
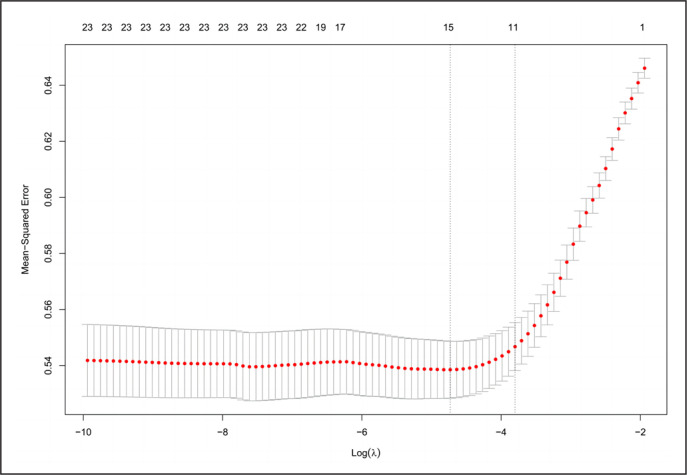
Plot of the Mean-Squared Error (MSE) and the number of technical performance variables in the model as functions of log(λ) for the cross-validation analyses. The red dots are the mean form the cross-validation and the bars indicate mean + 1SE and mean − 1SE, respectively. The minMSE + 1SE of minMSE (right) and minMSE (left) are indicated by the dashed vertical lines.

**FIG. 2:**
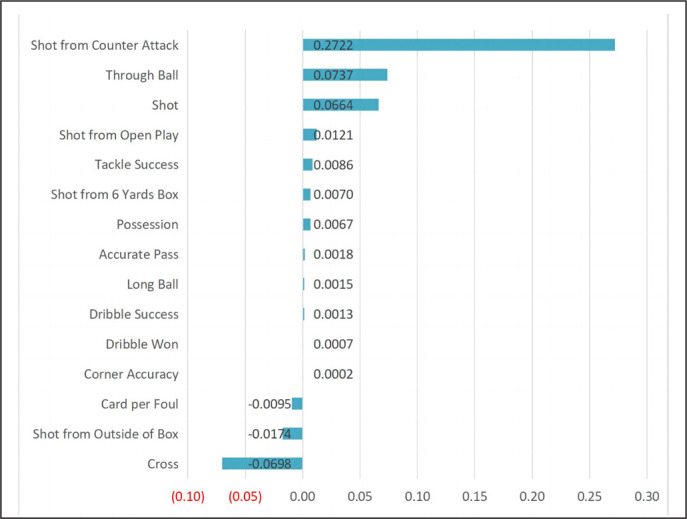
Regression coefficients for LASSO screening variables.

Figure 1 shows the number of technical performance variables for λ values of minMSE and minMSE + 1SE. We found that λ values of minMSE provided the following 15 technical performance variables: shot, shot from open play, shot from counter attack, shot from the 6-yard box, shot from outside of the box, long ball, through ball, cross, corner accuracy, accurate pass, possession, dribble success, dribbles won, tackle success, and cards per foul. These 15 technical performance variables were identified as important factors that could significantly influence match outcomes. Figure 2 shows the screened variables and their regression coefficients, ranked according to their importance regarding their impact on match outcomes. Descriptive statistics (mean ± SD) for these 15 technical performance variables are presented in Table 3.

**TABLE 3:** Descriptive statistics for fifteen technical performance variables.

Variables	n	Mean	SD	Min.	Max.
Shot	1166	12.82	5.723	1	35
Shot from Open Play	1166	9.14	4.443	0	29
Shot from Counterattack	1166	0.39	0.742	0	4
Shot from 6 Yards Box	1166	6.50	7.896	0	45
Shot from Outside of Box	1166	39.49	17.161	0	100
Long Ball	1166	58.38	13.828	15	120
Through Ball	1166	1.59	1.921	0	14
Cross	1166	16.45	8.548	0	62
Corner Accuracy	1166	45.20	29.766	0	100
Accurate Pass	1166	418.14	140.487	100	979
Possession	1166	50.00	12.079	19	81
Dribble Success	1166	60.02	13.846	0	100
Dribble Won	1166	10.49	4.721	0	33
Tackle Success	1166	62.67	11.197	16	100
Card per Foul	1166	16.52	11.335	0	71

The results showed that shot from counterattack had the greatest impact on match outcome, and a positive correlation was identified (regression coefficient, RC = 0.2722). Through ball, shot, shot from open play, tackle success, shot from the 6-yard box, possession, accurate pass, long ball, dribble success, dribbles won, and corner accuracy had positive impacts on match outcome (RC = 0.0737, 0.0664, 0.0121, 0.0086, 0.0070, 0.0067, 0.0018, 0.0015, 0.0013, 0.0007, and 0.0002, respectively). Cards per foul, shot from outside of the box, and cross were negatively correlated with match outcome (RC = -0.0095, -0.0174, and -0.0698, respectively).

### Influence of meteorological factors on technical performance

**FIG. 3:**
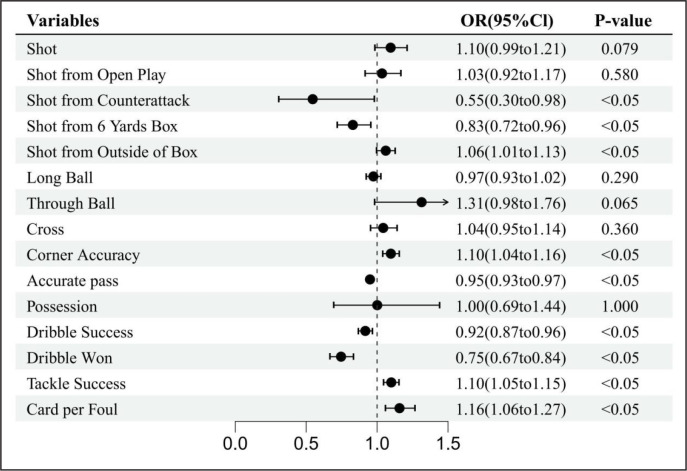
Forest diagrams illustrating the results of the effects of temperature on technical performance variables. Effects are shown as for each standard deviation increase in temperature, the odds that the variable will increase by 1 unit is (1-OR)*100%. OR odds ratio, Cl confidence interval.

**FIG. 4:**
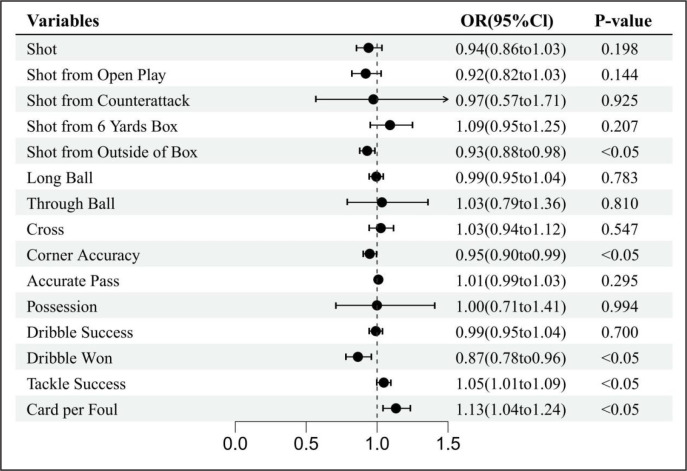
Forest diagrams illustrating the results of the effects of humidity on technical performance variables. Effects are shown as for each standard deviation increase in humidity, the odds that the variable will increase by 1 unit is (1-OR)*100%. OR odds ratio, Cl confidence interval.

**FIG. 5:**
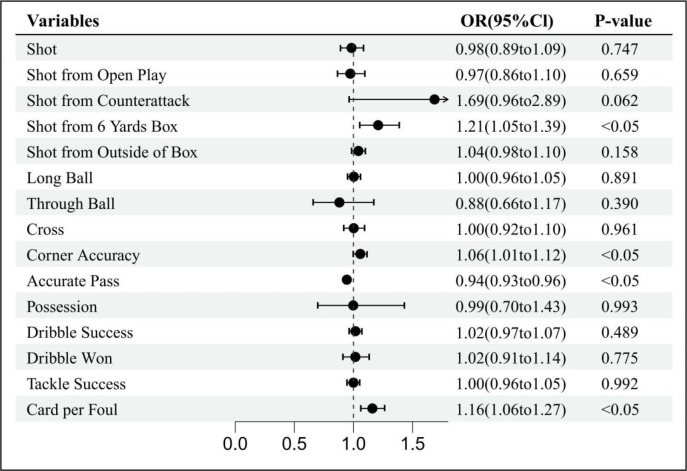
Forest diagrams illustrating the results of the effects of wind speed on technical performance variables. Effects are shown as for each standard deviation increase in wind speed, the odds that the variable will increase by 1 unit is (1-OR)*100%. OR odds ratio, Cl confidence interval.

Figures 3, 4, and 5 present the effects of air temperature, relative humidity, and wind speed on the technical performance variables. The results revealed that nine technical performance variables were significantly affected by air temperature. Air temperature had the most significant and negatively correlated effect on shot from counterattack, indicating that every unit increase in air temperature was associated with a 45% decrease in the odds of shot from counterattack (OR = 0.55, 95% CI: 0.30 to 0.98). Corner accuracy (OR = 1.10, 95% CI: 1.04 to 1.16), tackle success (OR = 1.10; 95% CI: 1.05 to 1.15), and shot from outside of the box (OR = 1.06, 95% CI: 1.01 to 1.13) were positively affected by air temperature, but only small effects were observed. Accurate pass (OR = 0.95, CI: 0.93 to 0.97) and dribble success (OR = 0.92, CI: 0.87 to 0.96) were also negatively affected by air temperature, but the odds were less than 10%.

The results also showed that dribbles won was significantly affected by air temperature and relative humidity; each unit increase in air temperature and relative humidity had a 25% (OR = 0.75, 95% CI: 0.67 to 0.84) and 13% (OR = 0.87, 95% CI: 0.78 to 0.96) odds of a decrease in the number of dribbles won, respectively. An increase in wind speed increased the odds of shot from the 6-yard box by 21% (OR = 1.21, 95% CI: 1.05 to 1.39), while an increase in air temperature decreased the odds of shot from the 6-yard box by 17% (OR = 0.83, 95% CI: 0.72 to 0.96). Cards per foul was the only variable that was significantly affected by air temperature, relative humidity, and wind speed. A unit increase in air temperature, relative humidity, and wind speed led to a 16% increase (OR = 1.16, 95% CI: 1.06 to 1.27), 13% (OR = 1.13, 95% CI: 1.04 to 1.24), and 16% (OR = 1.16, 95% CI: 1.06 to 1.27) increase, respectively, in the odds of cards per foul.

## DISCUSSION

The current study identified the key PIs that can significantly affect match outcome, and a predictive analysis was performed to quantify the impacts of meteorological factors on teams’ technical performance in the UEFA Champions League. The results showed that 1) shot, shot from open play, shot from counter attack, shot from the 6-yard box, shot from outside of the box, long ball, through ball, cross, corner accuracy, accurate pass, possession, dribble success, dribbles won, tackle success, and card per foul were the important technical PIs that significantly affected match outcome, and shot from counterattack had the greatest impact; and 2) air temperature, relative humidity, and wind speed had the most significant influence on shot from counterattack, dribbles won, shot from the 6-yard box, and cards per foul.

### Factors influenced by a single meteorological factor

The results revealed that most of the variables related to shot and offense (shot from open play, shot from counterattack, dribbles won, and possession) were important technical indicators that distinguished the match results (winning, drawing, and losing). This finding is consistent with those of previous studies [25, 17]. The LASSO regression results showed that shot from counterattack was the most important technical PI. This means that when there are more shots from counterattacks, the probability of winning the match is high. This trend is partially similar to the research conducted by Sarmento et al. (2018), who argued that teams that use counterattacking tactics compared with positional play were 40% more likely to win a game [26]. However, in the current study, we found that teams’ performance in counterattack was strongly influenced by air temperature. Previous studies have shown that higher air temperature reduced the high-intensity effort of players in all playing positions [1]. When a player or team made a counterattack, most of their behaviour was associated with high-intensity actions [27]; the higher the air temperature was, the lower was the number of counterattacks, with correspondingly less chance of counterattack shots. From a physiological perspective, counterattack in football is a short-term, highintensity action [28]. This action is characterized by anaerobic metabolism [29], and anaerobic exercise is largely influenced by air temperature [30]. Therefore, when the meteorological condition worsens, a more direct or faster attack may make it harder for the team to achieve the desired goal [31]. These findings highlighted the importance of considering the effects of air temperature on team performance during a match.

### Factors influenced by two meteorological factors

Contrary to the previous view that meteorological factors have little impact on a player’s technical performance [1], one new finding in this study was that both dribbles won and shot from the 6-yard box were significantly influenced by two meteorological factors. The reason for this difference may be attributed to the cumulative effect of player performance, implying that small differences in player technical performance can contribute to significant differences at the team level [2]. Our results show that when air temperature and relative humidity increase, the odds of dribbles won decrease. Previous studies have shown that playing football in hot and humid conditions increases the body’s sweating rate, leading to dehydration. Since electrolytes help to produce nerve impulses and transmit these impulses to the muscles and brain, imbalances in electrolyte concentrations caused by sweating can impair or reduce muscle, cognitive, and neurotransmission functions [32]. When a player’s cognitive function and reaction decrease, it can severely affect their judgment of movement and, consequently, dribble performance. This means that when the air temperature and relative humidity on the field are high, players should keep the ball for the shortest possible time.

It is well known that the higher the wind speed is, the greater is the ball and player resistance [33]. The current study demonstrated that the odds of increasing the number of shots from the 6-yard box with increasing wind speed was 21%. However, the trajectory of a football depends not only on wind speed but also on wind direction and other factors [34]. Since this study only considered wind speed indicators, it is not possible to confirm that a player’s willingness to shoot from close range is caused by the wind’s resistance to the ball. Another interesting finding of this study is that teams were more likely to try shooting from a distance when air temperature increased. This may be because an increase in air temperature leads to a warmer core temperature in the attacking players, making them prone to fatigue [12]. Consequently, the players do not have a strong desire to increase the passes or physically confront their opponents inside the 6-yard box. Depending on the air temperature at the time of the match, coaches should decide whether they want their players to play more long-range shots or pass into the 6-yard box to try scoring a goal.

### Factors influenced by three meteorological factors

“Cards per foul” has rarely been mentioned as an indicator in other articles. However, as an important indicator in this study that affects match outcome, it is influenced by air temperature, relative humidity, and wind speed. The findings showed that these three meteorological conditions increase the cards per foul.

In fact, researchers have demonstrated that climatic environments affect mood and cognition to a great extent, and air temperature is a major factor in this relationship [35]. A consequence of decreased positive affect in hot weather is the increase in aggressive acts of violence [36, 37]. During autumn and winter, when wind speed increases, people’s autonomous attention naturally decreases [38]. This means that as the air temperature and relative humidity increase, players will become more ‘aggressive,’ leading to more foul play and, therefore, higher likelihood of receiving a red or yellow card. As the referees are also on the football field during a match, their decisions are influenced by meteorology. When referees, both central and assistant referees, are in a mildly dehydrated state, their concentration and distance judgment are affected, which affects their decisions [39]. This can better explain why all the three meteorological factors affect “cards per foul.” Therefore, when air temperature, relative humidity, and wind speed increase, players must control their emotions and avoid physical violations. In future research, more meteorological variables such as altitude and site conditions should be considered. Similarly, future research should not be limited to variance analysis alone but should include predictive analysis.

The findings of the current study may help coaches and analysts to better understand the influences of meteorological factors on important technical variables during the evaluation of teams’ technical performance. However, the limitations of the current study should be noted. Firstly, only the statistics of technical actions and events during matches were analysed, while information on physical and tactical behaviours was not considered due to the availability of match data. This may have restricted the understanding of teams’ match performance. Secondly, situational variables were not included in this study, which could have led to a less comprehensive understanding of the influence of meteorological factors. Further studies should assess the interactive effects of meteorological factors. Exploring the impact of meteorological factors on more dimensions of technical performance and assessing more meteorological factors can be incorporated in future studies. Lastly, only technical variables and three meteorological variables were analysed in this study. More variables that can affect the team’s match performance, such as tactical and physical variables, and more meteorological factors, such as wind direction, should also be considered in future research to provide more valuable insights for coaches.

## CONCLUSIONS

In summary, most of the important technical performance variables in the UCL are shot-related and passing-related variables. Among these variables, shot from counterattack is the variable that was most significantly influenced by only air temperature. “Dribbles won” and “shots from the 6-yard box” were significantly influenced by a humid and hot environment while “shots from the 6-yard box” was also positively affected by wind speed. Cards per foul can be positively influenced by all three meteorological factors simultaneously.

This study emphasizes the need for coaches to pay close attention to changes in meteorology before and during each match. During matches, meteorological conditions changed dynamically; effective tactical adjustments should be made in the second half based on changes in any of these three factors.
